# The addition of arginine deiminase potentiates Mithramycin A-induced cell death in patient-derived glioblastoma cells via ATF4 and cytochrome C

**DOI:** 10.1186/s12935-023-02873-2

**Published:** 2023-02-27

**Authors:** Charlotte Linke, Thomas Freitag, Christin Riess, Jana Vanessa Scheffler, Katharina del Moral, Nina Schoenwaelder, Tomas Fiedler, Adina Fiebig, Philipp Kaps, Daniel Dubinski, Björn Schneider, Wendy Bergmann, Carl Friedrich Classen, Claudia Maletzki

**Affiliations:** 1 Department of Medicine Clinic III—Hematology, Oncology, Palliative Medicine, Rostock University Medical Center, Ernst-Heydemann-Str. 6, 18057 Rostock, Germany; 2 University Children’s Hospital, Rostock University Medical Center, Ernst-Heydemann-Straße 8, 18057 Rostock, Germany; 3 Institute of Medical Microbiology, Virology, and Hygiene, Rostock University Medical Centre, Schillingallee 70, 18057 Rostock, Germany; 4 Department of Neurosurgery, Faculty of Medicine, University of Rostock, Rostock, Germany; 5 Institute of Pathology, Rostock University Medical Center, Rostock, Germany; 6 Core Facility for Cell Sorting & Cell Analysis, Laboratory for Clinical Immunology, Rostock University Medical Center, 18057 Rostock, Germany

**Keywords:** Arginine auxotrophy, Senescence, Long-term treatment, 3D culture, Radiation, Stemness

## Abstract

**Background:**

Arginine auxotrophy constitutes a shortcoming for ~ 30% of glioblastoma multiforme (GBM). Indeed, arginine-depleting therapy using arginine deiminase from *Streptococcus pyogenes* (SpyADI) has proven activity against GBM in preclinical studies. The good safety profile of SpyADI renders this agent an ideal combination partner for cytostatic therapy.

**Methods:**

In this study, we combined the antineoplastic antibiotic Mithramycin A (MitA) with SpyADI to boost single-agent activity and analyzed underlying response mechanisms in-depth.

**Results:**

MitA monotherapy induced a time- and dose-dependent cytotoxicity in eight patient-derived GBM cell lines and had a radiosensitizing effect in all but one cell line. Combination treatment boosted the effects of the monotherapy in 2D- and 3D models. The simultaneous approach was superior to the sequential application and significantly impaired colony formation after repetitive treatment. MitA monotherapy significantly inhibited GBM invasiveness. However, this effect was not enhanced in the combination. Functional analysis identified SpyADI-triggered senescence induction accompanied by increased mitochondrial membrane polarization upon mono- and combination therapy. In HROG63, induction of lysosomes was seen after both monotherapies, indicative of autophagy. These cells seemed swollen and had a more pronounced cortically formed cytoskeleton. Also, cytochrome C and endoplasmatic reticulum-stress-associated proteins ATF4 and Calnexin were enhanced in the combination, contributing to apoptosis. Notably, no significant increases in glioma-stemness marker were seen.

**Conclusions:**

Therapeutic utilization of a metabolic defect in GBM along with cytostatic therapy provides a novel combination approach. Whether this SpyADI/MitA regimen will provide a safe alternative to combat GBM, will have to be addressed in subsequent (pre-)clinical trials.

**Graphical Abstract:**

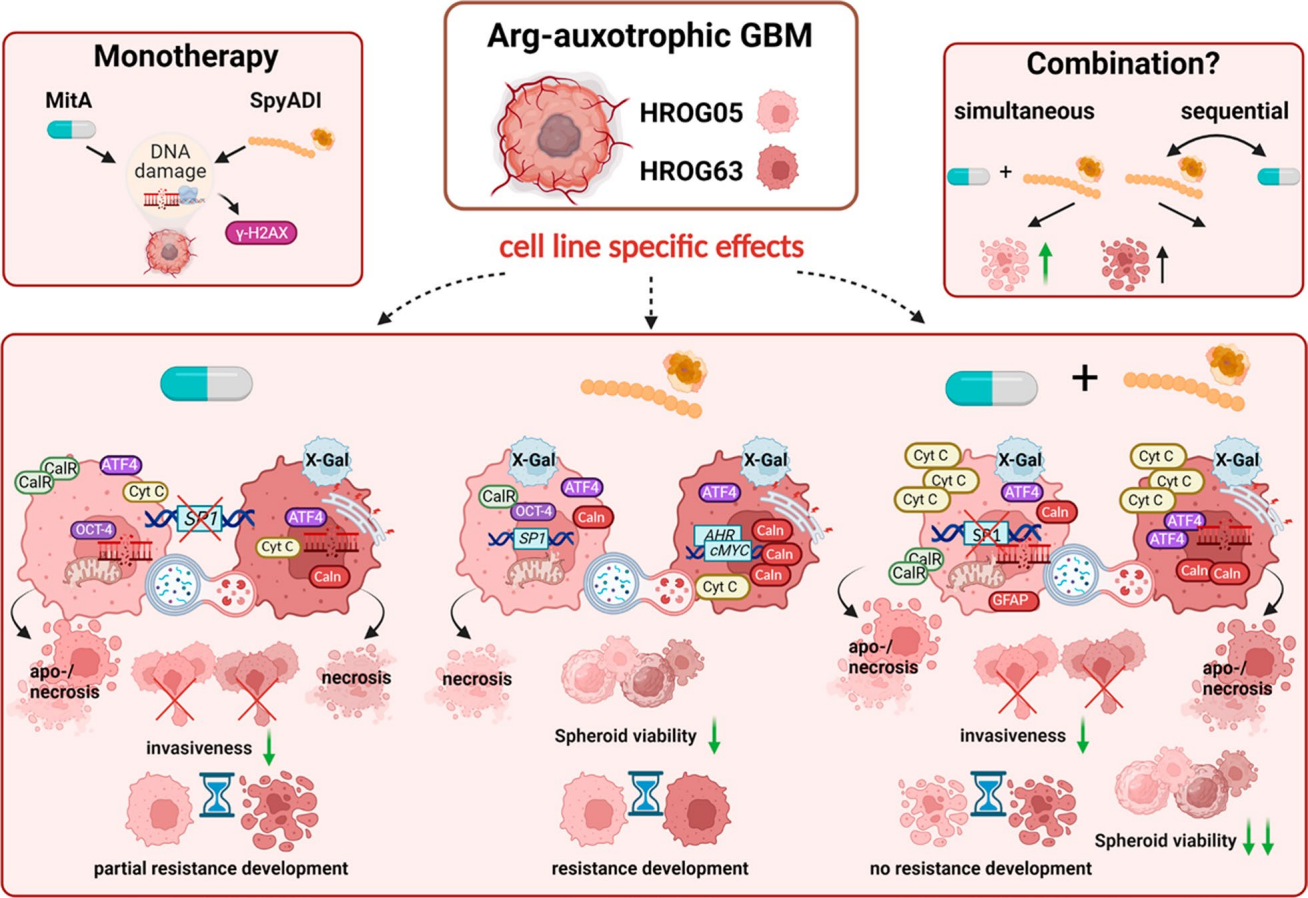

**Supplementary Information:**

The online version contains supplementary material available at 10.1186/s12935-023-02873-2.

## Introduction

Glioblastoma multiforme (GBM) is one of the most aggressive cancers found in humans and the most prevalent malignancy of the central nervous system [1–4]. GBM treatment includes surgery, chemo-/radiotherapy, and tumor-treating fields to interfere with the cancer cells’ dividing ability [5]. The latter showed improvement in survival comparable to that of a second round of chemotherapy. Owing to the high intratumoral heterogeneity and cellular plasticity, the overall prognosis remains extremely poor, and more effective treatment strategies are urgently needed.

Around 30% of all glioblastoma multiforme (GBM) cases are arginine auxotrophic [6–8]. This implies the dependence on exogenous arginine and constitutes a therapeutic target [9, 10]. Several preclinical and clinical studies described the successful application of enzyme-based arginine deprivation strategies [11–14]. As for GBM, we and others previously reported the elimination of Arg-auxotrophic GBM upon arginine depletion therapy in vitro and in vivo [7, 15–17]. Our group focused on the application of arginine deiminase from *Streptococcus pyogenes* (SpyADI) [16, 18]. Mechanistically, antitumoral effects of SpyADI were due to autophagy, senescence, and necrosis as well as altered gene expression in GBM. Stress-related genes, including superoxide dismutase (SOD) 1/2 were highly upregulated in SpyADI-treated GBM cells [17]. While these may represent a GBM-specific rescue strategy, the altered gene profile might sensitize for specific combination approaches.

A major element interacting with the proximal region of the SOD1 promoter is the transcription factor Sp1 (specificity protein). Sp1 is upregulated during transformation and a negative prognostic factor in several cancers, including GBM [19]. The antineoplastic antibiotic Mithramycin A (MitA) is an Sp1 inhibitor [20–22]. This agent binds to GC-rich sequences located in the minor groove of the DNA and blocks the binding of Sp1 to their GC-rich promoters. MitA additionally inhibits the transcription of several proto-oncogenes and is thus antineoplastic against testicular cancer, Paget's disease of bone, and chronic myeloid leukemia [23]. Another positive side effect is the reduction of hypercalcemia in cancer patients. However, the described hepatotoxicity, thrombocytopenia, and hemorrhagic diathesis may preclude the administration in high doses to reach clinical responses [24]. Still, the ability to cross the blood–brain barrier and enter the cerebrospinal fluid makes this compound an ideal combination partner for SpyADI-based strategies, whose good safety profile is well-documented [25–29]. In addition, low-toxic “mithralogues” developed for improved efficacy, regained interest in preclinical research [23].

Here, we hypothesized that using MitA as a combination partner prevented SpyADI-induced Sp1 upregulation. We were able to show that (I) simultaneous combination treatment is superior to sequential therapy in 2D- and 3D-GBM models; (II) this combination upregulates cytochrome C and ATF4, leading to apoptosis; and (III) the long-term treatment has improved antineoplastic activity.

## Material and methods

### Patient-derived GBM tumor cell lines and culture conditions

Patient-derived GBM cell lines (HROG02, HROG05, HROG52, HROG63, GBM03, GBM06, GBM14, and GBM15) were established in our lab from patients with primary (HROG02, HROG52, GBM03, GBM06, GBM14, and GBM15) or recurrent (HROG05, HROG63) GBM at WHO °4 (Table 1). Patient consent was obtained in all cases. All procedures were approved by the Ethics Committee of the *Rostock University Medical Center*, University of Rostock (Ethikkommission an der Medizinischen Fakultät der Universität Rostock, St.-Georg-Str. 108, 18055 Rostock, Germany; reference number II HV A 2009/34 and A 2018-0167) following generally accepted guidelines for the use of human material. Detailed information about the cell lines is given in [30]. Cells were cultured in 2D and 3D (HROG05 and HROG63) using Dulbecco’s Modified Eagle medium supplemented with the nutrient mixture F-12 containing 10% FCS, L-glutamine (6 mmol/l), and 1% of the antibiotics penicillin/streptomycin (all from Pan Biotech, Aidenbach, Germany). The incubation took place at 37 °C in a humidified atmosphere of 5% CO_2_. In the 2D experiments, GBM cells were seeded into 96- (short-term treatment), 24- (invasion assay), or 6- (long-term treatment) well plates (Greiner Bio-One, Kremsmünster, Austria). 3D-spheroids were induced by incubation for 72–96 h with full medium in 96-well ultra-low-attachment (ULA) plates (Greiner Bio-One, Kremsmünster, Austria).

**Table 1:** clinical data of GBM patients’ from which tumors were obtained for cell line establishment

Tumor case/cell line	Age/ Gender	Diagnosis/ Tumor location	MGMT promoter methylation status Molecular characteristics
HROG02	69/m	GBM WHO °4/ right-sided parietooccipital	Methylated
HROG05	56/f	GBM WHO °4/ left-sided temporal lobe	Methylated *Kras*^*G12D*^
HROG63	47/m	GBM WHO °4/ left-sided temporal lobe	Unmethylated
GBM03	81/m	GBM WHO °4/ right-sided temporal lobe	Unmethylated
GBM06	71/m	GBM WHO °4/ temporal lobe	−
GBM14	63/m	GBM WHO °4/ left-sided temporal lobe	Unmethylated
GBM15	40/m	GBM WHO °4/ left-sided temporal lobe	Unmethylated

*m* male,* f* female

### Mithramycin A (MitA) and* S. pyogenes* arginine deiminase (SpyADI)

The therapeutic effects of the agents MitA (Cayman Chemical, Michigan, USA) and SpyADI were examined. The Arginine Deiminase from *S. pyogenes* (SpyADI, 35 mU/ml)) was heterologously expressed in *E. coli DHα* and purified as described in [31]. For functional and combination assays, MitA was used at IC_20_ (4 nM). The cells were treated simultaneously (SIM) and sequentially (SEQ) for two therapy cycles (144 h) with MitA and SpyADI.

### Treatment protocols and viability assays

Cell viability of 2D-cultures was assessed by calcein acetoxymethyl ester (Calcein-AM) (Biomol GmbH, Hamburg, Germany) staining. GBM cells were seeded in plates and incubated overnight. To assess MitA susceptibility, cells were treated for 1 × 72 h or 2 × 72 h with increasing doses (ranging from 2 to 100 nM). Control cells were left untreated. Thereafter, Calcein-AM (4 mM) was added and incubated for 20 min (37 °C, 5% CO_2_). The fluorescence analysis was performed on a multiwell-plate reader (Tecan Reader Infinite M200, Tecan Group AG, Männedorf, Switzerland) at an excitation/emission of 485/535 nm. Additionally, ten cycles of long-term therapy (72 h each) were performed. The cells were treated in mono- and combination therapy, either SIM or SEQ. For the latter, cells received five cycles SpyADI followed by five cycles MitA. Cell viability was assessed by Calcein-AM assay and crystal violet (0.2%) staining (Sigma-Aldrich, St. Louis, USA). The viability of the 3D-spheroid cultures was analyzed using CellTiter-Glo 3D cell viability assay (Promega, Walldorf, Germany) following the manufacturer’s instructions. The luminescence signal was measured with the GloMax Microplate luminometer (Promega, Walldorf, Germany).

### γ-Irradiation

After 24 h of treatment, GBM cells were irradiated with 2 Gy using an IBL 637 (CIS Bio-International, Codolet, France). Following the irradiation, the medium was replaced by fresh medium, followed by 72 h incubation at 37 °C and 5% CO_2_. This treatment cycle was repeated once. Thereafter, cell viability was measured using the Calcein-AM assay. Double-strand breaks (DSB) were assessed with γ-H2AX staining in 8 Well chamber µ-slides (ibidi, munich, Germany) as described before [32].

### 2D- and 3D-Invasion-model

A modified Boyden chamber technique (Greiner Bio-One, Kremsmünster, Austria) with Matrigel-coated membranes (Corning, Amsterdam, Netherlands) was used to examine the invasive behavior of GBM cells after treatment. Before seeding, cells were cultured for 24 h in a serum-free medium. After that, cells were seeded with serum-free medium supplemented with the cytostatic agents in the Matrigel-coated inserts (ThinCerts, 8 µm, Greiner Bio-One). To stimulate the cells to migrate through the membrane, the chamber below was filled with full medium. After 72 h, invading cells were quantified by WST-1 staining (1:10 in serum-free medium, Merck KGaA, Darmstadt, Germany). The analysis was done after 3 h incubation using the Tecan Reader at an excitation/emission of 480/650 nm. To document the invasiveness of tumor spheroids after treatment, 96-ULA well plates (Greiner Bio-One, Kremsmünster, Austria) were placed on ice after 4 days of sphere formation; half of the medium was removed, and cytostatic drugs were added including EGF (1%, ImmunoTools, Friesoythe, Germany) to stimulate the invasion into U-bottom wells containing ice-cold matrigel (Corning, Amsterdam, The Netherlands). The spheroids were monitored for 10 days and images were taken on days 0, 5, and 10 using the Leica microscope DMI 4000B (Leica, Heidelberg, Germany).

### Immunogenic cell death, senescence and apoptosis/necrosis assay

GBM cells were treated for 72 h and stained for 30 min at 4 °C with an anti-Calreticulin-antibody (1:50, Cell Signaling Technology, Danvers, USA). Then, cells were stained with a secondary FITC-conjugated donkey-anti-rabbit antibody (1:50, BioLegend, San Diego, USA). Calreticulin translocation was quantified using the flow cytometer FACS Calibur (BD Biosciences, New Jersey, USA) at an excitation/emission of 495 nm/525 nm.

To detect senescent cells after treatment, β-Galactosidase staining was done. This staining detects the enzyme β-Galactosidase at pH 6, which is characteristic of senescent cells. A commercially available kit (Cell Signaling Technology, Leiden, The Netherlands) was used following the manufacturer’s instructions. The medium was removed and cells were washed, fixed in fixative solution (15 min, RT), and incubated with β-galactosidase staining solution at 37 °C overnight. The development of blue color as an indicator of senescent cells was analyzed by using a microscope. The number of senescent cells was quantified concerning the total cell number per high power field (HPF). Additional stainings for specific senescence markers were done using CDKN2A/p16INK4a antibody (JC8) (1:50, Santa Cruz), Alexa Fluor® 488 p21 Waf1/Cip1 (1:300, Cell Signaling), and Alexa Flour® 594 anti-p53 antibody (1:50, Biolegend) as described before [33].

For detecting apoptotic/necrotic cells, a flow cytometry-based assay was used as described before [32]. Briefly, early and late apoptotic cells were detected by either Yo-Pro-1 or Yo-Pro-1/propidium iodide (PI) positivity. Necrotic cells were defined as Yo-Pro-1 negative/PI positive.

### MMP & autophagy

The assays MitoTracker CMXRos (20 nM), LysoTracker Green DND-26 (50 nM), and ER-Tracker Blue-White DPX (500 nM) were prepared according to the manufacturer’s instructions (Cell Signaling Technology, Thermo Fisher Scientific). After 72 h of treatment, Mitochondria and ER were stained for 30 min at 37 °C, and slides were washed twice. Acidic lysosomes were stained prior to analysis. Images were taken using fluorescence microscopy (Leica DMI 4000B).

### ER stress and stemness marker

Cells were fixed with 2% paraformaldehyde (PFA) w/o methanol (15 min, Thermo Fisher Scientific, Darmstadt, Germany), permeabilized, and blocked with 0.5% Triton X-100 (Thermo Fisher Scientific, Waltham, USA) in 2% BSA (PAN-Biotech, Aidenbach, Germany) for 60 min. ER stress markers included: Alexa 647 anti-ATF-4 antibody (B-3, 1:50, Santa Cruz), Alexa 594 anti-calnexin antibody (AF18, 1:50, Santa Cruz), and Alexa 488 anti-cytochrome c (1:50, Biolegend). For stemness, antibody mixtures, either containing anti-GFAP (1:200, Alexa Fluor 594, BioLegend, San Diego, USA) and anti-A2B5 (1:200, Alexa Flour 647, BioLegend, San Diego, USA), or anti-Oct-4 (1:500, Alexa Fluor 647, BioLegend, San Diego, USA) and anti-Nanog (1:500, Alexa Flour 488, BioLegend, San Diego, USA) were added and staining was done at 4 °C overnight. The next day, GFAP/A2B5-antibody mix was stained with Phalloidin green (1:50, BioLegend, San Diego, USA). Nuclei were counterstained with DAPI (1:1.000, Biomol, Hamburg, Germany) and cells were analyzed using a Zeiss microscope Axio Observer 7 (Zeiss, Oberkochen, Germany).

### Spectral flow cytometry

Functional analysis was done by spectral flow cytometry using two in-house designed multicolour panels. Panel 1 was used to study apoptosis, necrosis, proliferation, and autophagy. Panel 2 examined viability, methuosis, and immune regulation. For this purpose 0.5 × 10^6^ cells were taken/panel and processed. All procedures were performed using staining buffer (PBS, 2 mM EDTA, 2% BSA).

Panel 1: Membrane permeabilization was done as first step (True Nuclear Transcription Factor Buffer Set, Biolegend, San Diego, California, United States, True-Nuclear™ 1X Fix concentrate, 45 min, RT). Then, the True-Nuclear™ 1X Perm Buffer (Biolegend, San Diego, California, United States) was added, cells were washed (350 × g, 5 min) and stained with antibodies for intracellular staining (in 100 µl True-Nuclear™ 1X Perm Buffer): V450 rat anti-histone H3 (1:40, BD Biosciences, Heidelberg, Germany), and PE/Cyanine7 mouse anti-H2A.X phospho (clone: 2F3, 1:40, Biolegend, San Diego, California, United States). Staining was done for 30 min at RT, reaction was stopped with True-Nuclear™ 1X Perm Buffer, followed by two washing steps (350 × g, 5 min). Cells were finally resuspended in 0.35 ml staining buffer.

Panel 2: Extracellular staining was done for 20 min at RT in staining buffer (in 100 µl): PerCP/Cyanine5.5 anti-human CD274 (1:62.5, Biolegend), PE/Cyanine7 mouse anti-human CD325 (1:25, N-Cadherin, clone: 8C11, Biolegend), and APC-Vio 770 mouse anti-human CD324 (1:15, E-Cadherin, clone 67A4, Biolegend). Afterwards, cells were washed two times followed by membrane permeabilization (BD Transcription Factor Buffer Set, BD, 1 × Fix/Perm Working solution, 45 min, 4 °C). Then, the 1 × Perm/Wash Buffer (BD) was added, cells were washed (350 × g, 5 min) and were stained with antibodies for intracellular staining (in 100 µl 1 × Perm/Wash Buffer): Alexa Fluor 647 mouse anti-human Glut1 (1:500, BD) and Alexa Fluor 700 mouse anti-human Glut4 (1:100, clone: # 925932, R&D, Minneapolis, Minnesota, USA). Staining was done for 30 min at room temperature, reaction was stopped with 1 × Perm/Wash Buffer, followed by two washing steps (350 × g, 5 min). Cells were finally suspended in 0.35 ml staining buffer.

All measurements were done on a spectral flow cytometer (Cytek Aurora, Cytek Biosciences, Fremond, California, United States) in the Core Facility for Cell Sorting and Cell Analysis, University Medical Center Rostock, Rostock, Germany.

### Quantitative real-time PCR

RNA was isolated using the RNeasy Mini Kit (Qiagen). 1 µg mRNA and 50 ng random Hexamer Primer (Bioron, Ludwigshafen am Rhein, Germany) were incubated for 10 min at 70 °C. Sample mixes were completed with 5 × RT buffer complete, dNTPs, and 200 units reverase. cDNA was synthesized for 120 min at 45 °C followed by inactivation of the reverase for 10 min at 70 °C. 25 ng cDNA were used for quantitative real-time PCR with the SensiFAST Probe Lo-ROX Kit (Bioline, Memphis, Tennessee, USA). Predesigned or in-house designed Taqman gene expression assays were used: 6-FAM-3'BHQ-1 *cMyc* (Hs00153408_m1), SP1: 5' HEX-TCGGGGGATCCTGGCAAAAAGAAACA-3’BHQ-1*,* for 3’-AAGACAGTGAAGGAAGGGGC-5’, rev 3’-GCCATACACTTTCCCACAGC-5’, AHR: 5' HEX-GAGCTTCTTTGATGTTGCATTAAAATCCTCCCCT-3'BHQ-1*,* for 3’- TAGGCTCAGCGTCAGTTACC-5’, rev 3’- CTGGCCTCCGTTTCTTTCAG-5’. Self-designed 6-FAM-3'BHQ-1 *b-Actin* was used to detect β-actin as a housekeeping gene. The reaction was performed in the light cycler Viia7 (Applied Biosystems, Foster City, USA) with the following PCR conditions: 95 °C for 10 min, 40 cycles of 15 s at 95 °C, and 1 min at 60 °C. All reactions were run in triplicates. The mRNA levels of target genes were normalized to mRNA levels of *b-Actin*. The expression level of each sample was considered by calculating 2^−ΔCT^ (ΔCt = Ct_target_ – Ct_Housekeeping gene_), followed by 2^−ΔΔCT^ quantification, taking values of untreated controls as calibrator.

### Statistics

All values are given as mean ± SEM or mean ± SD. Statistical evaluation was performed using GraphPad PRISM 8 software (GraphPad Software, San Diego, CA, USA). Each experiment was done in at least three independent biological replicates. To perform statistical evaluation, one-way or two-way ANOVA (Bonferroni’s or Tukey’s multiple-comparison test) was used. The criterion for significance was taken to be P < 0.05. Significant differences are marked as follows: * vs. control; # vs. monotherapy; $ vs. SEQ-combination. The bliss independence model was used for calculating effects in the combination approach (SIM vs. SEQ).

## Results

### MitA monotherapy induces cell death and resensitizes GBM cells to radiation

**Fig. 1:**
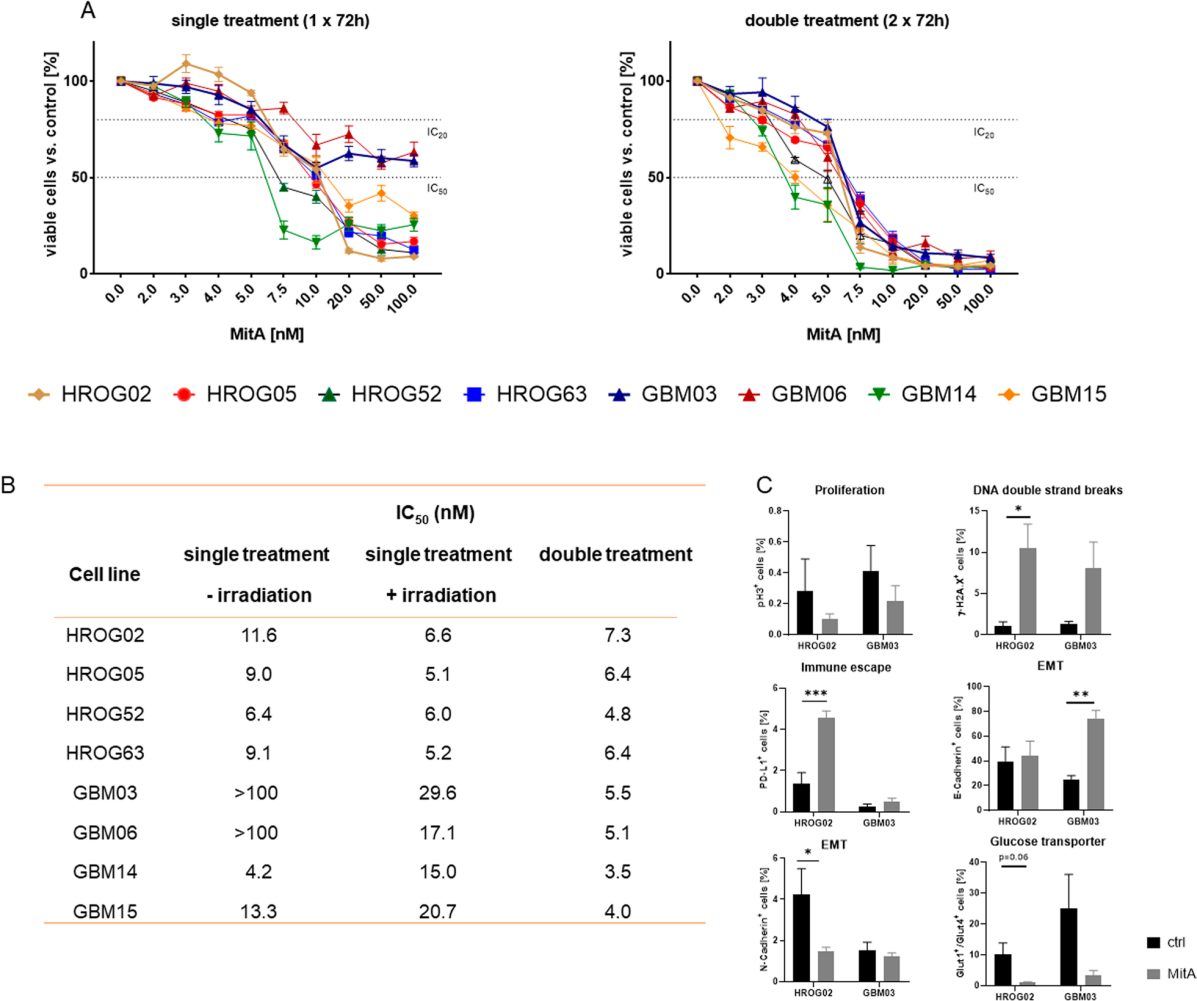
Viability assay using different MitA concentrations and influence on radiosensitivity. **A** Viability of GBM cell lines. HROG05 and HROG63 were treated with MitA for 1 × 72 h [red dots] or 2 × 72 h [blue dots] in ascending concentrations [2–100 nM], respectively. n = 3–7 independent experiments. Mean + SEM; IC_20_ and IC_50_ values are marked on the y-axis. **B** IC_50_ calculation for MitA after single (72 h) or double (2 × 72 h) treatment with ( +) or without (−) radiation. **C** Spectral flow cytometry. Analysis was done on HROG02 and GBM03 cells to dissect to potential differences in treatment response between ultra-low passage and long-term cultured cells. Mean + SD, n = 3 independent experiments. Two-way ANOVA (Tukey’s multiple comparison test)

Firstly, a panel of patient-derived GBM cell lines was screened for MitA sensitivity (Fig. 1A). This analysis revealed a time- and dose-dependent cytotoxicity, notably, in a nanomolar range (Fig. 1A). Effects were boosted in 8/8 cell lines after 2 × 72 h of treatment resulting in virtually complete cell death. In all cases, IC_50_ values were below 8 nM (Fig. 1B). To test whether similar mechanisms account for MitA sensitivity between ultra-low passage (< 20) and long-term cultured (> 40) cells, a comparative flow cytometry-based panel approach was done. GBM03 (ultra-low) and HROG02 (long-term) cells were included, because both cell lines have comparable population doubling times (~ 45 h) and in vitro morphology. The number of proliferating cells in G2-phase (= pH3^+^) was equally reduced in both lines, however, only HROG02 had significantly higher numbers of DNA double strand breaks. Figure 1C shows the numbers of γH2AX^+^ cells, indicating DNA damage. Also, the amount of PD-L1^+^ cells was only higher in HROG02, but not in GBM03 cells. To examine the potential involvement of epithelial-mesenchymal transition (EMT) as a cellular escape mechanism, classical EMT marker (E-Cadherin, N-Cadherin, Vimentin) were studied. Numbers of E-Cadherin-positive cells significantly increased in MitA-treated GBM03 cells, however, numbers of N-Cadherin-positive cells were only lower in HROG02 cells. In both cell lines, glucose transporters Glut1 and Glut4 were reduced after treatment (Fig. 1C).

Hence, we could show that different mechanisms contribute to MitA sensitivity. Freshly established ultra-low passage cell lines may have a lower vulnerability to drug-induced DNA damage and a low, but persistent, population of cells with EMT-characteristics.

### Radiosensitization by MitA monotherapy

**Fig. 2:**
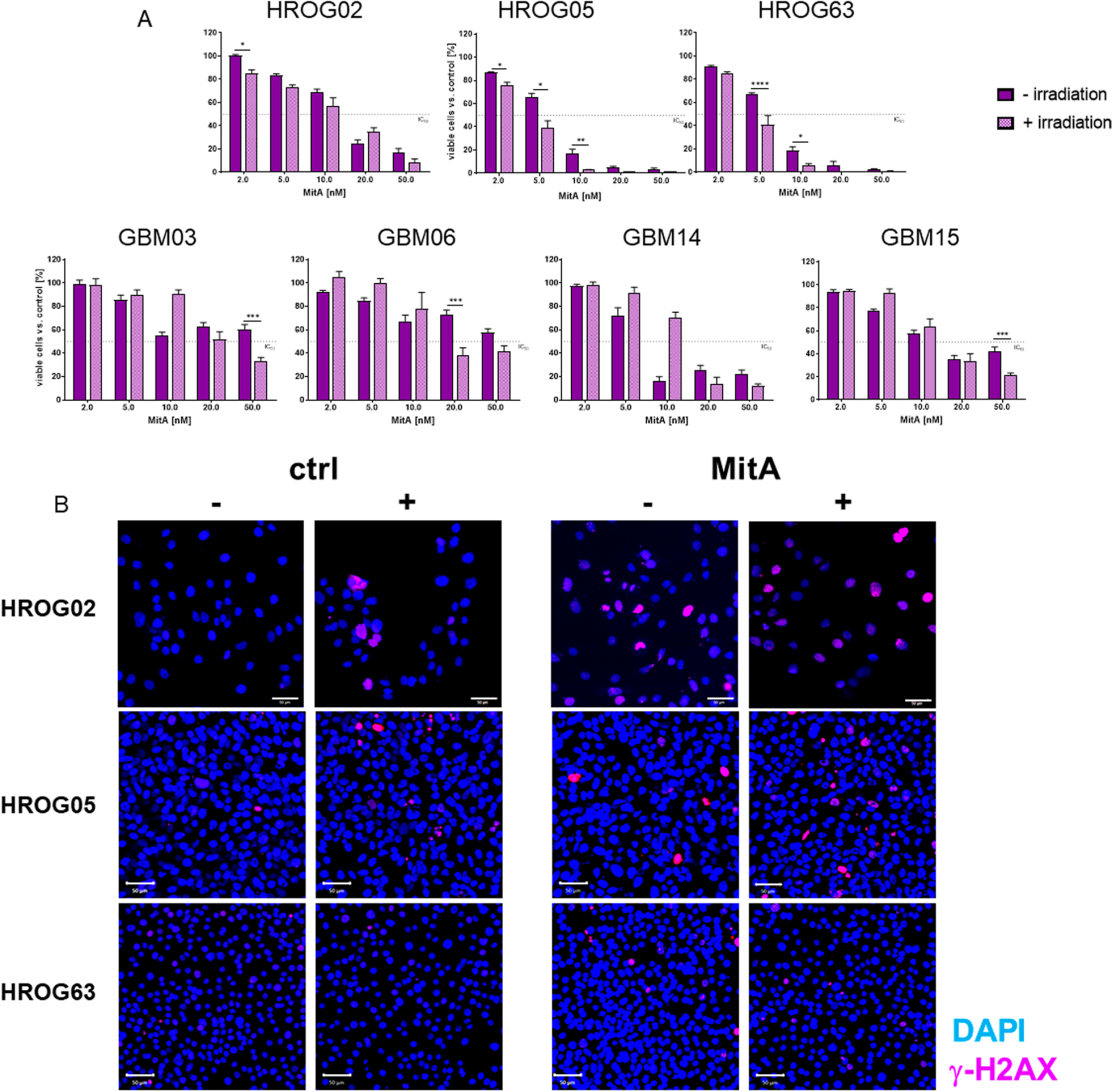
Effects of MitA on cellular radiosensitivity. **A** GBM cells were treated with increasing concentrations [2–100 nM] for 24 h before irradiation with 2 Gy (IBL637) and subsequent medium change [light purple] *vs*. non-irradiated MitA-treated cells [dark purple] for 2 × 72 h. Quantitative analyses were done using Calcein-AM assay. n = 3–7 independent experiments. *p < 0.05, **p < 0.01; ***p < 0.001; ****p < 0.0001 vs. control, Two-way ANOVA (Tukey’s multiple comparison test). **B** Representative images of 2D-cultured GBM cells (HROG02, HROG05, HROG63) stained with anti-H2A.X Phospho (Ser139) [red], treated with 5 nM MitA or left untreated with [ +] and without [−] irradiation. Scale bar as indicated: 50 µm. Nuclei were counterstained with DAPI. Images were taken on a Zeiss Elyra 7 Confocal Laser Microscope. Representative images of n = 3 independent experiments

Then, we studied the radiosensitizing effect of MitA (Fig. 2). Radiosensitization was confirmed in 6/7 GBM cell lines at different concentrations (Fig. 2A). In HROG02 and HROG05, this effect was visible at 2 nM, resulting in significantly lower cell viability after MitA and radiation (Fig. 2A). Notably, in the ultra-low passage GBM cell lines, radiosensitizing effects were only visible after higher MitA doses.

To check whether the reduced viability after radiation is attributable to radiation-induced DNA double-strand breaks, γ-H2AX immunostainings were done (Fig. 2B and Additional file 1: Fig. S1). Low-dose MitA treatment in combination with irradiation enhanced the number of γ-H2AX foci in most GBM cell lines. No differences were seen between long-term cultured (HROG02, HROG05, HROG63) and ultra-low passage cells (GBM03, GBM06, GBM14, GBM15).

On a basis of this preliminary result, a combination approach was done with the IC_20_ of MitA (4 nM) in the following analyses.

### Combination therapy of MitA and SpyADI inhibits cell growth significantly and boosts effects after long-term treatment

**Fig. 3:**
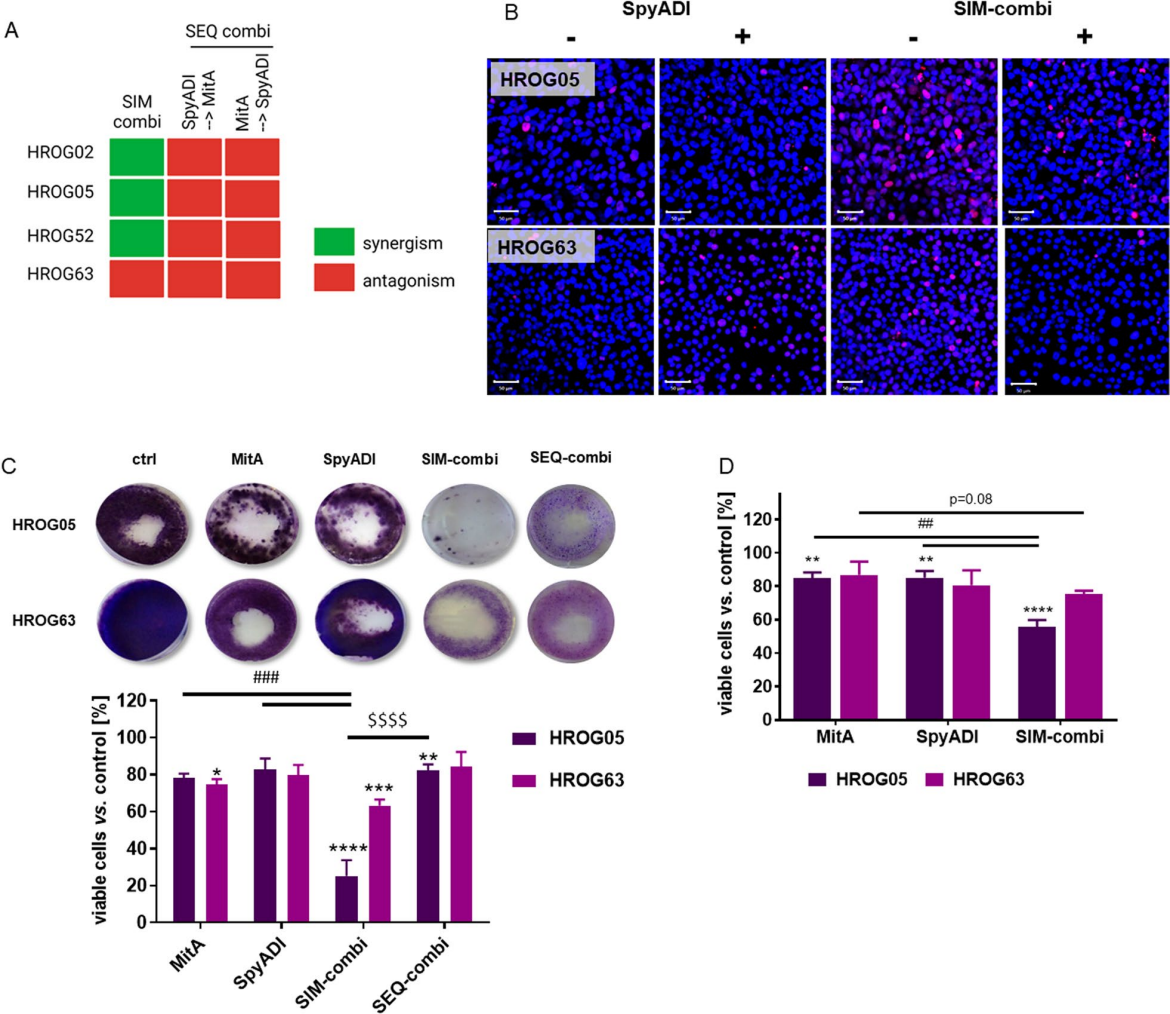
Cytotoxic effects of MitA and SpyADI combination. **A** Bliss independence calculation after combination treatment of GBM cell lines HROG02, HROG05, HROG52, and HROG63. Synergistic effects were obtained in 3/4 cell lines by applying the SIM combination approach. **B** γ-H2AX staining [red] of HROG05 and HROG63 treated with SpyADI and SIM combination with [ +] and without [−] radiation. Scale bar as indicated: 50 µm. Nuclei were counterstained with DAPI. Images were taken on a Zeiss Elyra 7 Confocal Laser Microscope. Representative images of n = 3 independent experiments. **C** HROG cells received 10 × 72 h consecutive mono- and SIM combination therapy cycles and stained with crystal violet to identify long-term treatment effects. Viability reduction (%) after treatment (HROG05: dark purple; HROG63: light purple) was quantified by normalization to control values (untreated cells, set to be 100%). n = 3 independent experiments. *p < 0.05, **p < 0.01; ***p < 0.001; ****p < 0.0001 vs. control; #### p < 0.0001 vs. monotherapy; $$$$ p < 0.0001 vs. SEQ-combination, Two-way ANOVA (Tukey's multiple comparisons test). **D** Viability in the 3D model in mono- and SIM combination regimens (HROG05 [black], HROG63 [grey]) was assessed with CellTiter-Glo 3D. n = 3 independent experiments. **p < 0.01; ****p < 0.0001 vs. control; ##p < 0.01 vs. monotherapy, Two-way ANOVA (Tukey's multiple comparisons test)

Next, we examined the effects of combined MitA and SpyADI treatment on four arginine-auxotrophic GBM cell lines (HROG02, HROG05, HROG52, HROG63). Combinations were applied simultaneously (= SIM) and sequentially (SEQ), the latter starting with SpyADI in the first line or the other way around. This analysis revealed a synergistic effect in 3/4 cell lines after simultaneous treatment (Fig. 3A). By contrast, the SEQ approach was always antagonistic.

To address this further, two cell lines were included in supplementary γ-H2AX stainings either with or without radiation. HROG05 and HROG63 cells were chosen, since these two cell lines showed differential responses upon SIM-combination treatment (Fig. 3B). With this analysis, the maintenance of the radiosensitizing effects was confirmed in HROG05 cells (Fig. 3B). In HROG63 cells, no radiation-induced DNA double-strand break increase was seen, hence, the positive effects of MitA may vanish when SpyADI is given simultaneously.

To investigate the effects after long-term exposure and to identify potential early-developing resistance mechanisms, the two GBM cell lines HROG05 and HROG63 were included in further experiments. We focused on these cells because of their high sensitivity to MitA after short-term treatment. Besides, these two cell lines were established from recurrent GBM cases, raising the question of whether MitA may constitute an alternative approach for 2nd or 3rd line treatment.

Both cell lines received 10 consecutive therapy cycles of MitA, SpyADI, or the combination (Fig. 3C). MitA or SpyADI monotherapy slightly reduced the cell viability by ~ 20% (*vs*. control). In the SIM-combination, the number of viable cell colonies was significantly reduced (vs. control and the respective monotherapy). Again, HROG05, molecularly characterized by a *Kras*^*G12D*^ mutation and MGMT promoter methylation (vs. HROG63: Kras^wt^, MGMT unmethylated), was most vulnerable to the combination treatment. Using a direct comparison between the SIM- and SEQ combination supported the superiority of the former. Consequently, we then focused on this regimen in the more clinically relevant 3D cell culture model (Fig. 3D). Even in this condition, spheroids were susceptible to both agents, either alone or in combination. In HROG05, the number of viable cells decreased by approximately 50% when MitA and SpyADI were combined. In HROG63 the effects were weaker, still, spheroid viability was reduced, especially in the SIM-combination (p = 0.08 vs. control).

### MitA counteracts SpyADI-induced *SP1* and *cMyc* expression

**Fig. 4:**
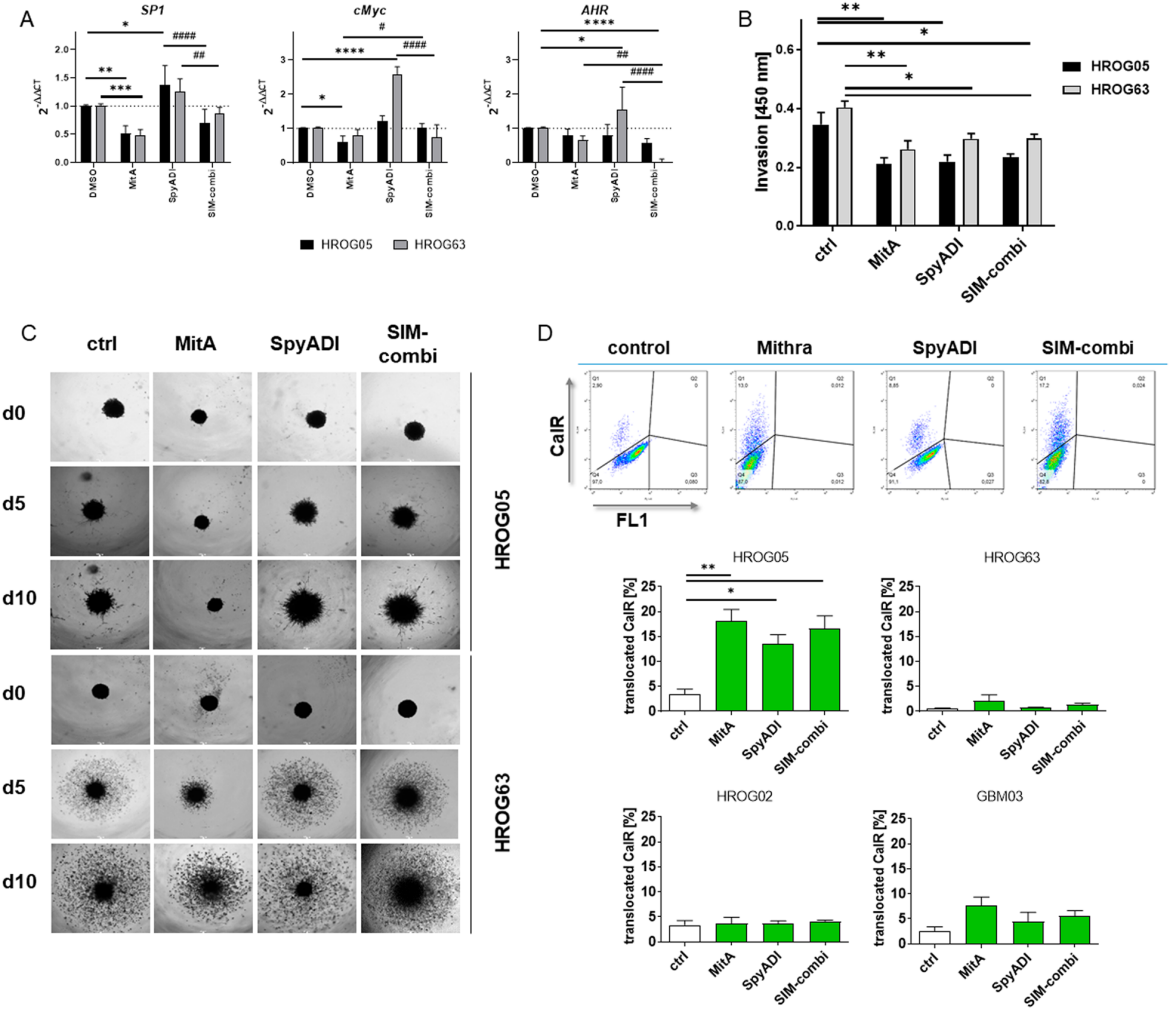
Gene expression changes, reduced invasion, and CalR translocation after MitA mono- and combination therapy. **A** Quantitative qPCR was conducted with cDNA from control cells and after treatment upon reverse transcription of total RNA as described in material and methods. Analysis was done in triplicates with n = 5 independent experiments. All data are given as 2^−ΔΔCT^ values + SD. *p < 0.05, **p < 0.01 vs. control, #p < 0.05 vs. control; ***p < 0.01 vs. control; ****p < 0.0001 vs. control; #p < 0.05 vs. monotherapy, ##p < 0.01 vs. monotherapy ###p < 0.001 vs. monotherapy, ####p < 0.0001 vs. monotherapy, Two-way ANOVA (Tukey’s multiple comparisons test). **B** Effects of MitA and its combination on cell invasion (HROG05 [black], HROG63 [grey]) assessed by a Boyden chamber assay [absorbance: 450 nm]. Mean + SEM, n = 3 independent experiments. *p < 0.05, **p < 0.01 vs. control, One-way ANOVA (Tukey’s multiple comparison test). **C** Representative images of MitA-treated 3D-cultured GBM cell invasion into a Matrigel matrix (scale bar: 250 μm). GBM spheres were monitored for a total of 10 days and images were taken on day 0, 5 and 10 using the Leica microscope DMI 4000B. Representative images of n = 3 independent experiments. **D** CalR translocation was detected after 2 × 72 h mono- and SIM combination treatment by staining CalR on the cell surface. Upper part: representative dots plots showing CalR-positive cells. Lower part: Quantitative analysis. 10,000 events were measured and the percentage [%] of cells showing CalR translocation were provided. n = 3 independent experiments. *p < 0.05, **p < 0.01 vs. control, One-way ANOVA

MitA is a known SP1 inhibitor. Hence, we analyzed *SP1* expression levels in HROG05 and HROG63 cells under treatment. MitA monotherapy and the SIM combination significantly decreased *SP1* expression in HROG05 and HROG63 cells (Fig. 4A). Vice versa, SpyADI upregulated *SP1*, but this was effectively counter-regulated in the SIM combination to values comparable to controls.

Then, we checked for additional target genes and focused on *cMYC* and the aryl hydrocarbon receptor (*AHR).* The former is a proto-oncogene with different effects on tumor cells and the latter plays a central role in tolerogenic immunity to promote GBM tumorigenesis [34, 35]. Both genes were significantly higher in SpyADI-treated HROG63 cells. Adding MitA suppressed gene expression, thus confirming the beneficial role of this agent in GBM treatment. Notably, the expression of *AHR* was completely inhibited under combinational treatment. In HROG05 cells, no significant changes were seen, suggesting a minor relevance of these genes in treatment response.

### MitA mono- and combination therapy reduce the invasiveness in 2D and 3D models

Then, the ability of cells to migrate from the toxic environment of MitA, SpyADI, and the SIM combination was investigated in HROG05 and HROG63 cells. A modified Boyden chamber was used to study invasion in 2D cultures (Fig. 4B). All treatments significantly reduced the invasiveness (*vs*. control). Effects were comparable between the three treatments, notably in HROG05 and HROG63 cells. By transferring this approach to the 3D-spheroid invasion assay, microscopic evaluation for 10 days revealed substantial inhibition by MitA (Fig. 4C). Compared to the control, only a few HROG05 cells invaded the surrounding matrix after 10 days. In HROG63 cells, this effect was weaker, cells slowly started to invade the matrix from day five on. Monotherapy with SpyADI as well as the combination therapy did not affect the invasiveness of GBM cells in the spheroid model.

Hence, the inhibitory effect on GBM cell invasiveness by MitA was not enhanced in the SIM combination approach.

### Influence on immunogenic cell death, senescence, autophagy, and mitochondria

Immunogenic cell death (ICD) was quantified by calreticulin (CalR) translocation (Fig. 4D). Here, both cell lines responded differently. MitA and SpyADI monotherapy, as well as the combination induced CalR translocation in HROG05 cells. Highest values were measured after MitA monotherapy, reaching 18% CalR positivity. In HROG63, a slightly induced CalR translocation was detectable after MitA monotherapy, still only 2% of all cells were CalR positive. By contrast, neither SpyADI nor the SIM combination triggered CalR translocation in HROG63 cells. Hence, we assume selective alterations in HROG63 cells that may prevent the emission of immunogenic signals per se. To study this further, two additional cell lines (HROG02, GBM03) were included and ICD was studied upon treatment. In HROG02, no CalR translocation was detectable, while in ultra-low passage GBM03 cells, MitA evoked ICD (Fig. 4D). Here again, no increase was seen in the SIM-combination.

**Fig. 5:**
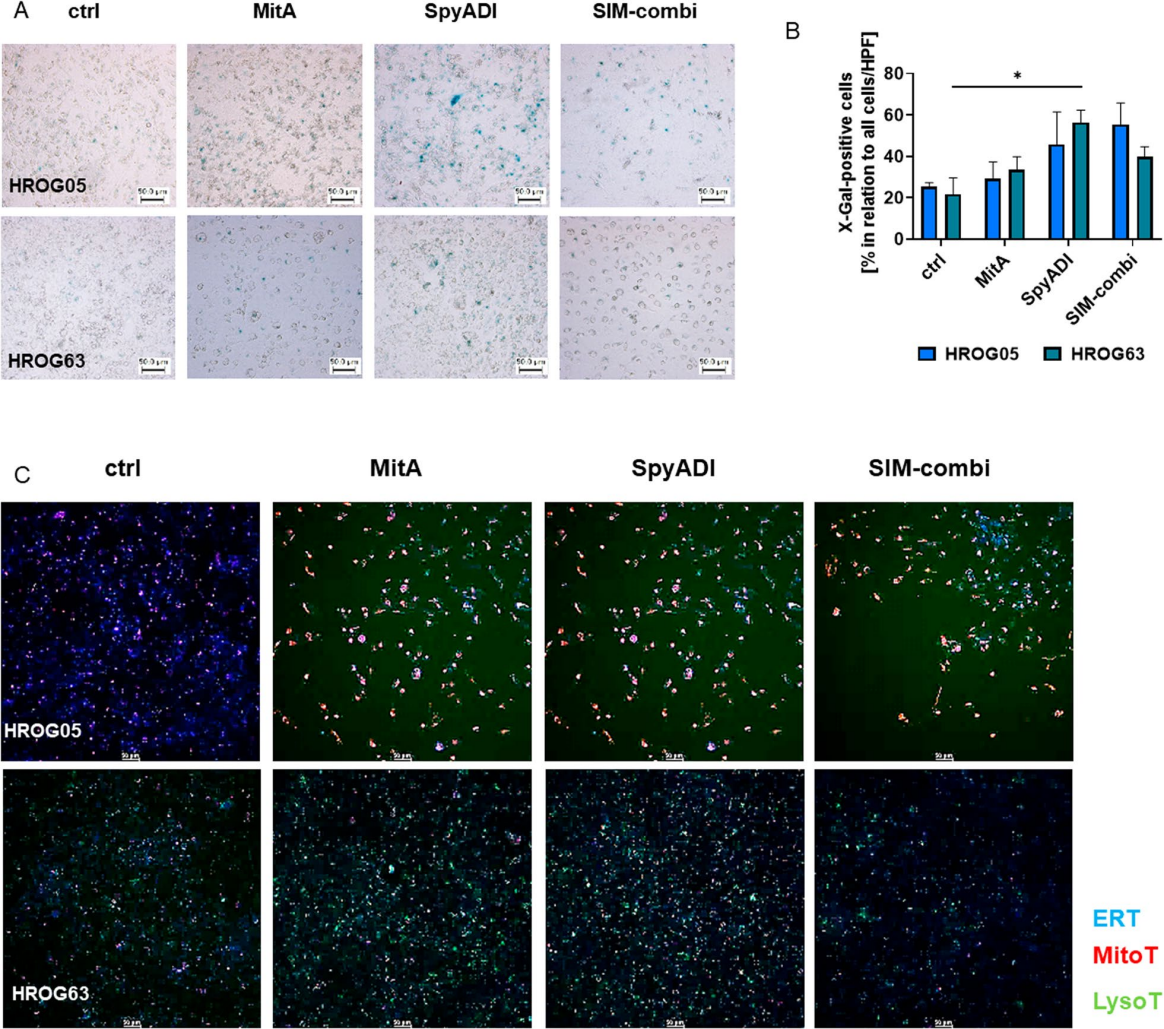
Effects of MitA mono- and combination therapy on senescence and cell stress. **A** Senescence was assessed by X-Gal staining. GBM cells (HROG05, HROG63) were treated with test substances for 2 × 72 h, fixed, and stained with β-galactosidase staining solution overnight at 37 °C without CO_2_. [blue: ß-galactosidase activity, indicative for senescence]. Representative images are shown (scale bar: 50 μm). **B** Quantitative analyses of X-Gal-positive cultured cells in relation to the whole-cell number/image [HROG05: blue, HROG63: green]. Mean + SEM, n = 3 independent experiments. * p < 0.05 vs. control, One-way ANOVA (Tukey’s multiple comparison test). **C** The effect on the mitochondrial activity, the lysosome formation, and the ER was determined after treatment for 2 × 72 h and staining with MitoTracker [red], LysoTracker [green], and ER-Tracker [blue] (scale bar: 50 μm, Leica microscope DMI 4000B). Representative merged images are shown, n = 3 independent experiments

Then, cellular senescence was investigated. Representative images of β-galactosidase staining are shown in Fig. 5A. Senescence was predominantly visible under SpyADI mono- and combination treatment, as indicated by higher numbers of blue spots (*vs*. control, Fig. 5A, 5B). The addition of MitA did not boost the effect of SpyADI with X-Gal-positive cell numbers being comparable to the monotherapy. To confirm X-Gal analysis, more specific senescence markers p53, p21, and p16 were examined (Additional file 2: Fig. S2). An upregulation of p16 and a slight increase of p21 and p53 was detected in HROG05 cells, notably in all treatments. In HROG63, marginal changes were detectable, but only after MitA or SpyADI monotherapy.

For functional analyses, we checked the influence on mitochondria, lysosomes, and endoplasmatic reticulum (ER). Representative images are shown in Fig. 5C. In HROG05, the mitochondrial membrane polarization (MMP) increased upon MitA and SpyADI monotherapy (*vs*. control). The effect was also preserved in the SIM combination. We additionally identified a reduced ER formation and a marginally induced lysosome formation in all treatments. In HROG63, no effect on the MMP or the ER could be demonstrated. Instead, induction of lysosomes was seen after both monotherapies, which was again not enhanced in the SIM combination (Fig. 5C). Also, no significant increase of LC3B was seen (*data not shown*), suggesting a minor role of autophagy in this setting.

In conclusion, the included cell lines showed individual cellular responses. in HROG05, the treatments mainly showed an effect on mitochondria, while in HROG63, induction of stress-induced lysosomes was evident.

### No treatment-associated changes in GBM stemness

**Fig. 6:**
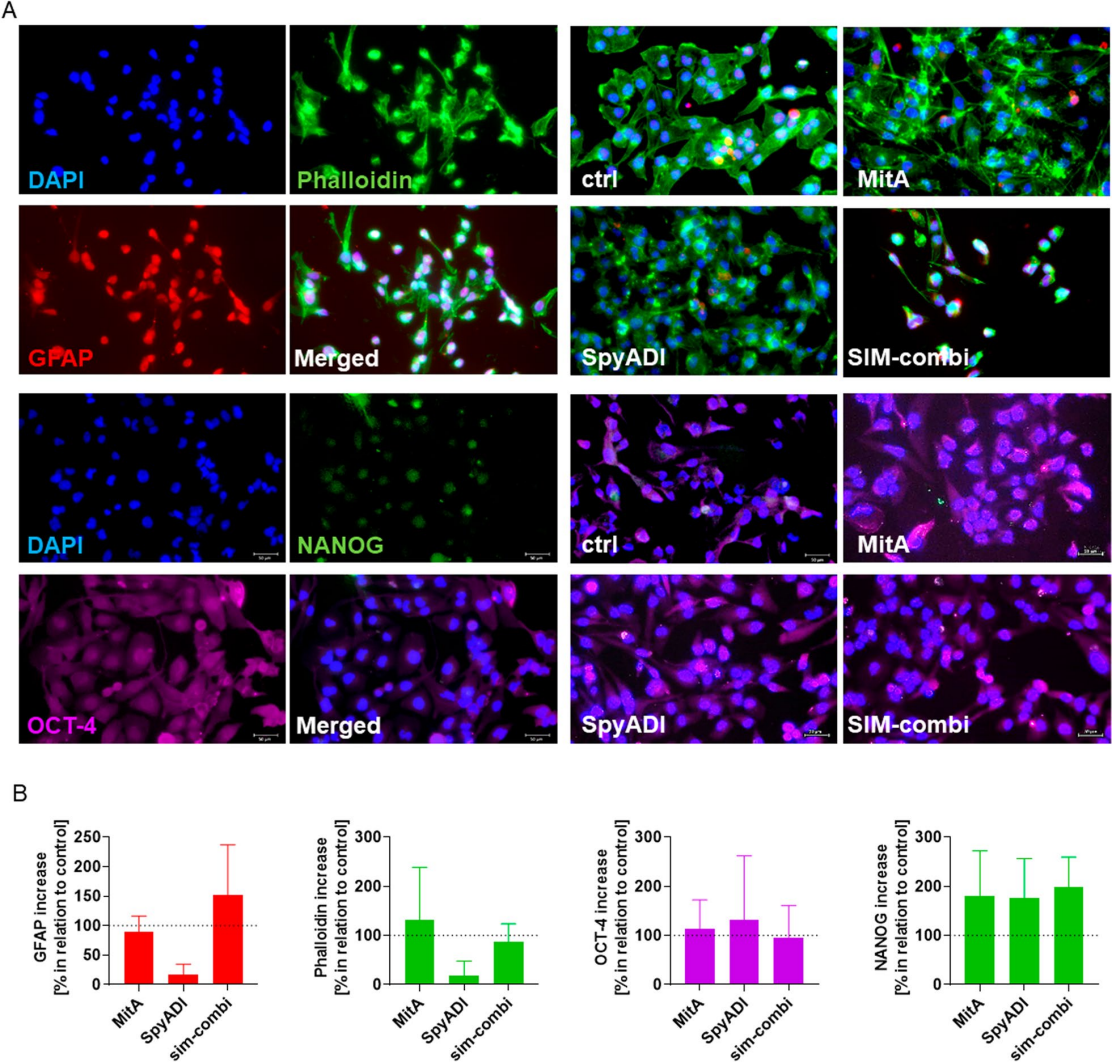
Pluripotency and self-renewal of HROG05 cells. **A**, **B** Cells were treated, fixed, permeabilized and stained with Phalloidin green, Alexa Fluor 594 GFAP [red], Alexa Fluor 647 Oct4 [purple], Alexa Flour 488 Nanog [green] and counterstained with DAPI. Images were taken on a Zeiss Elyra 7 Confocal Laser Microscope. **A** Representative single channel and merged images are shown. **B** Quantitative analysis of specific markers in relation to the control (= 100%). Mean + SEM, n = 3 independent experiments

Stem cell factors, such as OCT-4 and NANOG, are critical for pluripotency and the ability to self-renew embryonic cells and are also thought to play a role in GBM development and recurrence [36, 37]. A2B5 and GFAP are markers for neuroglial stem cells [38]. Here, we analyzed the abundance of these stem cell markers and additionally examined the impact on the cytoskeleton (Fig. 6). For this analysis, HROG05 cells were chosen because these cells showed the best response to our treatment approaches. SpyADI monotherapy marginally reduced the abundance of GFAP, MitA had no impact on this stemness marker. Likewise, no significant changes were visible in the SIM combination. Still, we identified changes in the cytoskeletal structure under treatment, especially after SpyADI monotherapy (Fig. 6A). When compared with controls, the cells seemed swollen and the cortically formed cytoskeleton was more pronounced. A2B5 remained undetectable under treatment. Additional stemness-marker OCT-4 and NANOG remained unchanged under treatment (Fig. 6B).

### MitA and SpyADI trigger apoptotic cell death via Cyt C and ATF4 expression

**Fig. 7:**
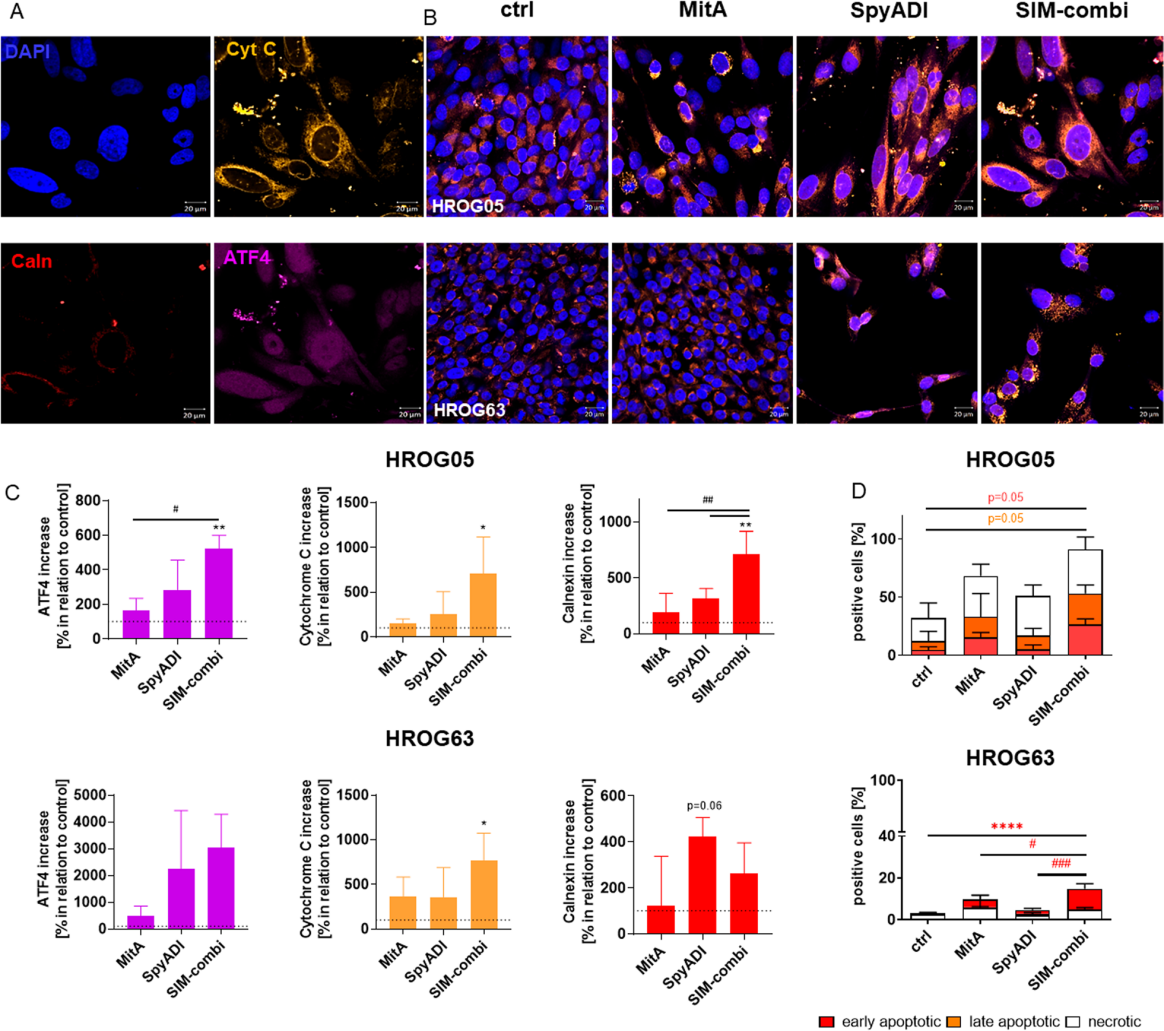
Influence on Cytochrome C and ER stress marker and flow cytometric quantification of apoptosis/necrosis. **A**–**C** To investigate the effect of the test substances on cytochrome C distribution and specific ER markers, cells were treated for 2 × 72 h and stained with Alexa Flour® 546 anti-Cytochrome C Ab, Alexa Flour® 594 anti-Calnexin Ab [red], and Alexa Flour® 647 anti-ATF4 Ab (scale bar: 50 μm). Nuclei were stained with DAPI. **A**, **B** Representative single channel (**A**) and merged (**B**) images are shown, n = 3 independent experiments. Images were taken on a Zeiss Axio Observer 7 microscope. **C** Quantification of fluorescence intensity using ImageJ software. n = 3 independent experiments. Given is the percentage increase in fluorescence intensity in relation to controls; *p < 0.05, **p < 0.01 vs. control, #p < 0.05 vs. monotherapy; ##p < 0.01 vs. monotherapy, One-way ANOVA. (D) Flow cytometry for detecting early (Yo-Pro-1 +) and late apoptotic (Yo-Pro-1 + /PI +) or necrotic (PI +) cells. Results show data from at least 3 independent experiments. Mean + SD. ****p < 0.0001 vs. control, #p < 0.05 vs. monotherapy; ###p < 0.001 vs. monotherapy, One-way ANOVA (Tukey’s multiple comparison test)

Cytochrome C controls both cellular energy metabolism and apoptosis. When freely present in the cytosol, it contributes significantly to the initiation of apoptosis. It also serves as a prognostic factor for GBM patients [39]. Besides, the ER stress markers ATF4 and Calnexin likewise trigger apoptosis. Here, we investigated the change in the expression of these three markers under the therapy (Fig. 7A–C). All treatments resulted in an increase in cytochrome C, ATF4, and Calnexin compared with control cells. Notably, SpyADI performed better than MitA in monotherapy. These changes were further enhanced in combination therapy. The only exception was seen in HROG63, where the combination reduced Calnexin abundance, compared to monotherapy with SpyADI.

To test whether the above changes in protein levels are related with apoptotic cell death, flow cytometry was done (Fig. 7D). With this analysis, increased numbers of early and late apoptotic cells (defined by Yo-Pro1 + and Yo-Pro + /PI +) were detected. Notably, apoptotic cell numbers were highest in the combination, even reaching significance in HROG63 cells (*vs.* ctrl and monotherapy). Hence, the induced cytotoxic effects of the mono- and combination therapy involve pro-apoptotic mechanisms mediated by ATF4, Calnexin, and re-induction of cytochrome C.

## Discussion

The poor prognosis of GBM raises the need for improved treatment strategies. Here, we investigated the efficacy of the Sp1 inhibitor MitA and the arginine deiminase SpyADI in mono and sequential/simultaneous combination therapy. Therefore, a panel of cell-based assays was employed to identify the best strategy and to unravel the underlying cellular mechanisms.

We could show that low-dose MitA treatment resensitizes GBM cells to irradiation, inhibits cellular invasion, triggers immunogenic cell death, and alters gene expression. Given the fact that MitA is a highly toxic agent per se, combination agents should be safe, with little or no severe toxic side effects. Hence, SpyADI may be an ideal combination partner for MitA. SpyADI is effective in the treatment of Arg-auxotrophic tumors [26, 40–44] with a very good safety profile [25, 45, 46]. We and others already proved the antitumoral activity against GBM [7, 15–17, 31, 33]. Mechanistically, SpyADI induces autophagy and senescence, inhibits invasion, and alters gene expression [17]. *Sp1* is one of the target genes that is most altered upon SpyADI. Hence, specific inhibition of this gene may further increase the cytotoxic effect of SpyADI. Indeed, by adding MitA to arginine-auxotrophic GBM cells, such promising boosted effects were seen here. Notably, the SIM combination was similar to or even better than the SEQ-combination approach.

In GBM, radiotherapy often fails because of the high ability to repair damaged DNA [47]. One possible explanation is the increased expression of *Sp1*. The latter is a transcription factor that influences many cellular processes, including DNA repair and tissue remodeling [48, 49]. Among other proteins, it controls the expression of ATM. This protein is an important cell cycle checkpoint kinase; thus, it functions as a regulator of a wide variety of downstream proteins, including tumor suppressor proteins p53 and BRCA1, checkpoint kinase CHK2, checkpoint proteins RAD17 and RAD9, and DNA repair protein NBS1. Increased expression of these damage control proteins mediates resistance to radiation-induced apoptosis and chemotherapies. Inhibition of Sp1 can overcome this resistance as described before in vitro and in vivo for different tumor entities, including lung and urothelial cancer [50]. In this study, MitA sensitized GBM cells to radiation at very low doses. Notably, a radiosensitizing effect was confirmed in 6/7 patient-individual cell lines. The only exception was GBM14, a cell line established from a male 63-years old patient with primary GBM. Mechanistically, MitA blocks GC-rich regions within the DNA and impedes Sp1 binding to this region. As a result, the transcription of the downstream DNA repair genes does not occur. In addition to Sp1 inhibition, MitA also causes the downregulation of Sp1 levels in cells by proteasome-dependent degradation [51] and directly via suppression of *Sp1* expression itself [52]. The latter was confirmed here upon low-dose MitA exposure to GBM cells. Vice versa, SpyADI led to an upregulation of Sp1, which was counter-regulated in the combination. Hence, the downregulation of downstream targets can be expected. Here, we focused on *cMYC* and *AHR*. The proto-oncogene *cMYC* was upregulated by SpyADI and reduced by MitA. Notably, *AHR* was significantly downregulated, but only in the combination approach. Since *AHR* and *cMyc* cooperate in the regulation of cellular metabolism [53], the downregulation of both genes in combination explains our findings of a boosted cytotoxic effect best. Indeed, targeting the metabolism of GBM cells is promising [54, 55].

Focusing on cellular effects, we observed reduced invasive activity upon MitA mono- and combination treatment of HROG05 and HROG63 cells, likely also attributable to the inhibition of Sp1. Still, the inhibition of invasion was not confirmed in the clinically relevant 3D models. Here, only MitA alone delayed invasion into the matrix.

To unravel the type of cell death induced by the treatments, we focused on 2D cultures. We demonstrated that all three treatment regimens evoked immunogenic cell death in HROG05, whereas marginal effects were observed in HROG63. Selective alterations that may prevent the emission of immunogenic signals per se may provide an explanation for the observed differences. Indeed, by including two other GBM cell lines (HROG02 and GBM03), MitA evoked ICD only in one cell line whereas no changes were seen in the other case. This adds to the fact that GBM is a non-immunogenic tumor per se, tumor cells barely undergo ICD. Still, MitA may provide a novel avenue for second-line cell-based immunotherapies in selected patients. In support of this, PD-L1 was higher after MitA treatment in one case with ICD-resistance.

Another potentially involved mechanism is cellular senescence. This was detectable after SpyADI treatment, but not enhanced in the combination, likely because of the high cytotoxicity of MitA that omits induction of complex cellular programs, such as senescence. Also, functional analyses of mitochondria, lysosomes, and ER revealed massively altered cell morphology in residual cells. Mitochondrial impairment was characterized by elevated mitochondrial membrane potential, mito-ROS, and vacuole formation, especially in HROG05 cells. This was accompanied by a reduced ER formation and a marginally induced lysosome formation in all treatments.

In HROG63, the mechanisms leading to cell death were different, which is also in line with our previous findings of a very individual response of all GBM cell lines [32, 33]. Here, increased lysosomes indicative of autophagy were observed [33]. Notably, cytochrome C was elevated in all treatments. Under basal conditions, cytochrome C was low in GBM cells, which is in line with their advanced malignancy state and may constitute an intrinsic resistance mechanism [39]. Knowing that cytochrome C inhibits tumor growth and triggers mitochondrial-mediated apoptosis, re-induction of this molecule upon MitA-SpyADI provides another piece of evidence for the high efficacy of our novel combination approach. Indeed, cytochrome C was just recently proposed as a prognostic biomarker [39]. Worth mentioning in this context is the fact that both cell lines had elevated levels of cytochrome C upon treatment, irrespective of the dominating mode of cell death, i.e. apoptosis or autophagy.

Another clinical challenge is the outgrowth of normally quiescent stem-like cells. This small and heterogeneous population within GBMs is the main reason for treatment failure and finally relapse. Hence, targeting stem-like cells is promising to move forward. Stem-like maintenance is driven by transcription factors, such as c-Myc, SOX2, NANOG, or OCT4 and is controlled by extracellular signaling pathways, super-enhancers, epigenetic regulation, and microRNAs. In our study, minor changes were seen in stem-like populations. One previous study reported increased CD133 expression levels in glioma-stem-like cells upon Sp1 overexpression that was suppressed by MitA [56]. Functionally, MitA binding to methyl-DNA-binding proteins contributed to the repression of transcription. Also, Chang et al. identified Sp1 induction in response to cellular stress that finally mediated TMZ resistance [57]. This group even proposed Sp1 inhibition to prevent GBM recurrence after primary therapy. Although we did not analyze stem-like expression in detail, we anticipate comparable mechanisms upon MitA treatment in our GBM models. In line with this, no significant changes in the stem-like phenotype were seen. The slight alterations after monotherapy were effectively counter-regulated in the combination. Quite in line, no resistance development was seen after ten consecutive treatment cycles. Effects were more pronounced in HROG05 compared to HROG63 cells, which were generally more vulnerable to our treatment approach. Still, amounts of cell colonies were significantly reduced especially when MitA was given simultaneously with SpyADI. Hence, the combination prevented resistance development even after long-term treatment.

To sum up our findings, we could show that low-dose MitA treatment effectively kills GBM cells via induction of DNA double-strand breaks, prevention of epithelial-mesenchymal transition, and triggering apoptosis. Owing to the high heterogeneity of GBM per se, we also identified differences between individual cell lines in terms of treatment sensitivity and the resulting effects on immunogenic cell death, migration, and invasion. The differences between individual cells may be attributable to (I) the origin, i.e. primary (HROG02, HROG52, GBM03, GBM06, GBM14, GBM15) or relapse (HROG05, HROG63), the MGMT promoter methylation status (methylated vs. unmethylated), and molecular alterations, i.e. HROG05 cells harbor a *Kras*^*G12D*^ mutation and MGMT promoter methylation—neither is detectable in HROG63. Still, the fact that both cell lines were established from recurrent GBM cases upon combined radio-/chemotherapy (radiation, Temozolomide [17]), but remained sensitive to MitA encourages additional studies for 2nd or 3rd line GBM treatment with this agent.

Besides, we could show that the addition of SpyADI as a metabolism-interfering agent boosted the effects of the monotherapy in most cases after simultaneous treatment. MitA-based combinations may not trigger stem-like characteristics in GBM, providing another rationale for future therapeutic interventions.

This study has several limitations: (I) all experiments were done in vitro without using an animal model and (II) no cytokine-release or functional immunological assays were done to study the relevance of immunogenic cell death on T cell stimulation capacity in GBM patients. This should be addressed in future research to test whether combined MitA/SpyADI treatment provides a safe alternative to combat GBM.

## Supplementary Information

**Additional file 1: Figure S1.** Extended radiosensitivity testing on ultra-low passage GBM cell lines. Representative images of 2D-cultured GBM cells (GBM03, GBM06, GBM14, GBM15) stained with anti-H2A.X Phospho (Ser139) [red], treated with 5 nM MitA or left untreated with [+] and without [-] irradiation. Scale bar as indicated: 50 µm. Nuclei were counterstained with DAPI. Images were taken on a Zeiss Elyra 7 Confocal Laser Microscope. Representative images of n = 3 independent experiments.

**Additional file 2: Figure S2.** Senescence detection via activation of p16/p21/p53. Representative images of GBM cells (HROG05, HROG63) demonstrate an increase in p16, p21 and p53 after MitA-related treatment regimes. The cells were treated as indicated, fixed, permeabilized, and stained with p21 Waf1/Cip1 (12D1) rabbit mAb (Alexa 488 conjugate) [green], p16 antibody (JC8): sc-56330 Alexa 546 [orange] and Alexa Flour® 594 anti-p53 antibody [red] (scale bar: 50 μm).

## Data Availability

The datasets generated during and/or analyzed during the current study are available from the corresponding author on reasonable request.
