# Supplementary figures and images for: The addition of arginine deiminase potentiates Mithramycin A-induced cell death in patient-derived glioblastoma cells via ATF4 and cytochrome C
Source: Cancer Cell Int. 2023 Feb 27;23:38. doi: 10.1186/s12935-023-02873-2 (PMC9969664; doi:10.1186/s12935-023-02873-2)

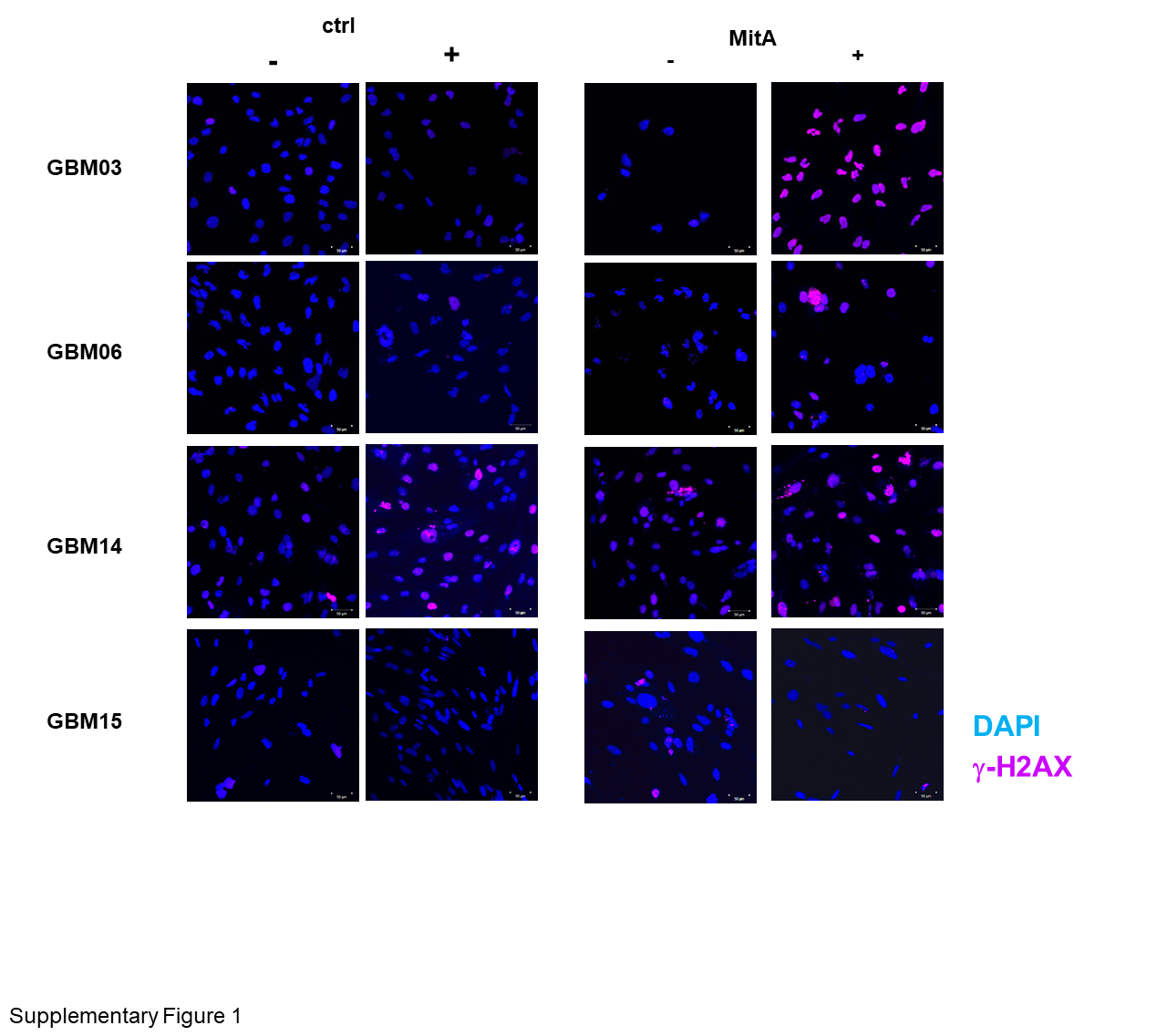

Supplement: Supplementary file 1 — Additional file 1: Figure S1. Extended radiosensitivity testing on ultra-low passage GBM cell lines. Representative images of 2D-cultured GBM cells (GBM03, GBM06, GBM14, GBM15) stained with anti-H2A.X Phospho (Ser139) [red], treated with 5 nM MitA or left untreated with [+] and without [-] irradiation. Scale bar as indicated: 50 µm. Nuclei were counterstained with DAPI. Images were taken on a Zeiss Elyra 7 Confocal Laser Microscope. Representative images of n = 3 independent experiments. [file 12935_2023_2873_MOESM1_ESM.tif]

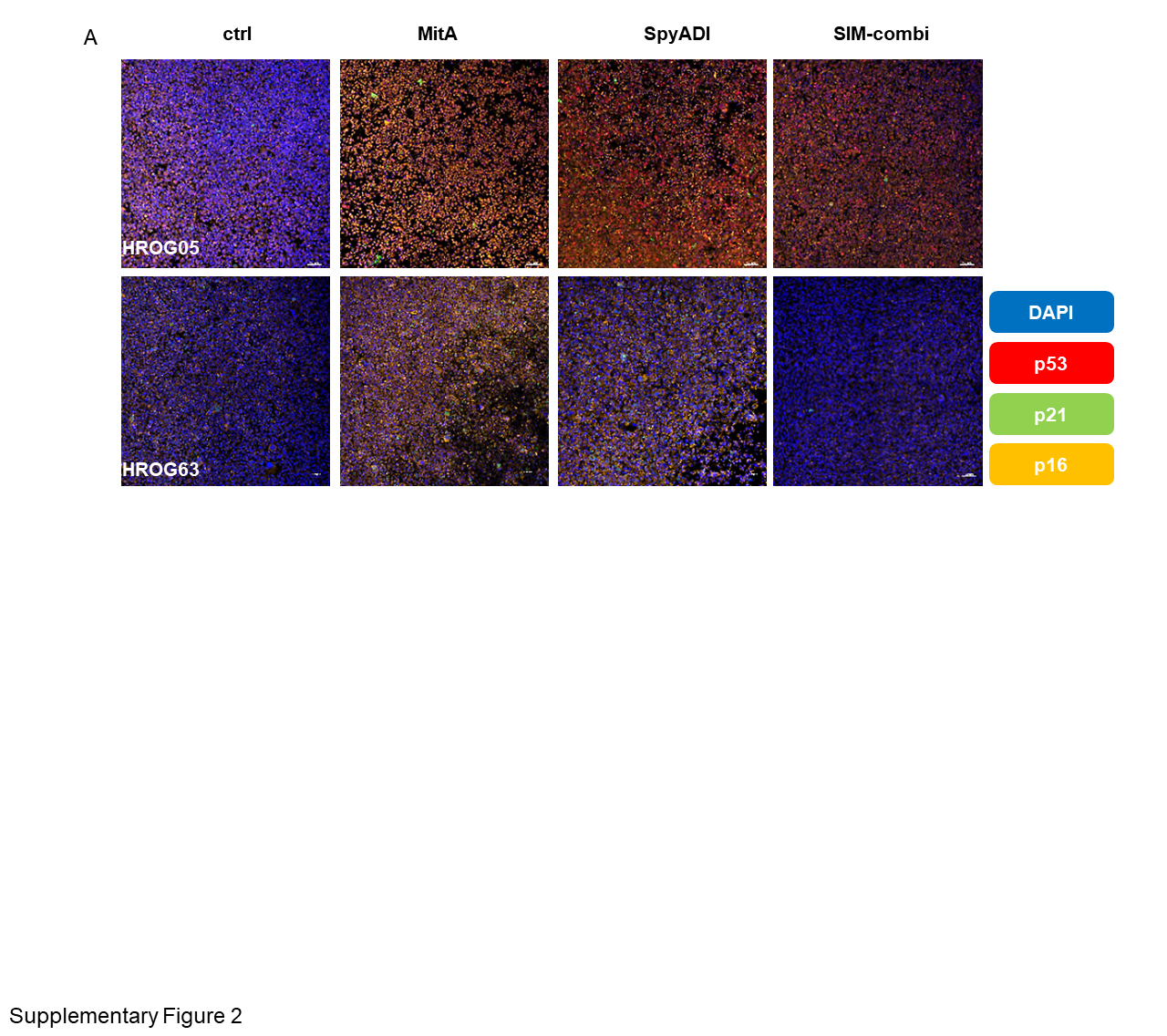

Supplement: Supplementary file 2 — Additional file 2: Figure S2. Senescence detection via activation of p16/p21/p53. Representative images of GBM cells (HROG05, HROG63) demonstrate an increase in p16, p21 and p53 after MitA-related treatment regimes. The cells were treated as indicated, fixed, permeabilized, and stained with p21 Waf1/Cip1 (12D1) rabbit mAb (Alexa 488 conjugate) [green], p16 antibody (JC8): sc-56330 Alexa 546 [orange] and Alexa Flour® 594 anti-p53 antibody [red] (scale bar: 50 μm). [file 12935_2023_2873_MOESM2_ESM.tif]
